# Supplementary material for: An interpretable framework for investigating the neighborhood effect in POI recommendation
Source: PLoS One. 2021 Aug 5;16(8):e0255685. doi: 10.1371/journal.pone.0255685 (PMC8341514; doi:10.1371/journal.pone.0255685)
Supplement: Attachment — Submitted filename: Yuan-Response-Letter.pdf [file pone.0255685.s002.pdf]

An Interpretable Framework for Investigating Neighborhood Effect in POI Recommendation

Guangchao Yuan[1], Munindar P. Singh[2], Pradeep K. Murukannaiah[3]

1 Microsoft, Mountain View, CA, USA
2 North Carolina State University, Raleigh, NC, USA
3 Delft University of Technology, Delft, The Netherlands

* P.K.Murukannaiah@tudelft.nl

Response Letter

Thanks to the editor and the reviewers for very helpful comments. We have addressed all the comments in this revision.

In this letter, we identify the page and line numbers of the revised manuscript (clean version), where we made pertinent changes corresponding to each comment.

We are also uploading a PDF file with tracked changes, created using *latexdiff*, which highlights the changes between the original submission and the revision.

Reviewer #1

The manuscript describes a new method to incorporate the neighborhood information when predicting a user's preference of a point-of-interest. The overall writing is fine and the authors have explained the functions used in different features.

Few comments are listed below:

- A bit of confusing about the number of times a user visits a POI. At lines 148-149, the authors said that "... not the exact number if the user visited the POI more than once.", but in Table 2, $n_{u,l}$ is the number of times a user u visits POI l. Please clarify.

  We address this comment in lines 227–228.

- Lines 160–162, the size of a neighborhood is the number of POIs within a distance, e.g. 100 m, 200 m, 500 m. This doesn't equal to the description at lines 161-162. There might be cases that the distance is larger, but no POIs are included.

  We address the comment in line 165, deleting the original statement.

- Lines 202–204, is there any reason why a word has to be appeared at least four times in a city? For example, why not five times?

  We use a heuristic approach to select the thresholds, as described in lines 208–211.

- Line 421, is the method "IRenMF"? The subtitle is the same as line 430.

  We address the comment in line 439.

- Lines 463–464, "... the embedding size of users (POIs) is 16 ...", should it be 32, as indicated at line 463?

  We address the comment in lines 481–483, making the statement clearer.

- Please indicate the meaning of bold text in tables, are they the best values in the corresponding categories?

  We address the comment for Table 3 (line 327) and Table 5 (line 485). We had already indicated the meaning of bold text in Table 4 (line 364).

Reviewer #2

This manuscript addresses a problem of recommending potential points of interest (POIs) to users. The particular emphasis is on a phenomenon called the ”neighborhood effect” that relates to a user’s preference for POIs in the neighborhood of a POI whose preference is already established. The authors accomplish two tasks. First, they identify features potentially related to the neighborhood effect and employ both logistic regression and FastTree to evaluate combinations of features. Second, they develop an artificial neural network (ANN) model meant to take advantage of the neighborhood effect and evaluate it against some established matrix factorization techniques and a simplified ANN. At a high level, the research seems good and the results correct, but I do have some detailed comments and thoughts to address.

- First, I marked up some required changes in red on the manuscript and scanned the result to a PDF, which is attached. Note that ”data set” is properly two words, though this is often abused.

  Thanks for the detailed mark up in the scanned PDF file. We addressed all the marked-up changes.

- Second, many of the references are on the older side; the manuscript would be improved by refreshing the References list with more recent papers.

  We updated the references, in Section 2.1 and in the Introduction (line 8), with more recent works than we had originally.

- Third, on page 5, the manuscript makes an offhand remark about a frequency distribution being a power law. This kind of casual remark is often made without justification, as is done here. It is likely the distribution is not a true power law in a statistical sense, so it is best to delete this claim.

  We deleted this claim.

- Fourth, there is little to no justification for the ANN architecture (Section 6.1); it is just stated as a fact. This approach is common in ANN research and does not reflect well on the area. Answer why you made the architectural decisions you did in a deeper way.

  We added more justifications in Section 6.1.

- Fifth, the Problem Formulation (Section 3) is merely two sets of questions. There is no real statement of computer science problems, where the inputs and outputs (solutions) are mathematically specified so that the authors and the readers can know when a problem is solved. I note that the authors are all computer scientists, so they know how to mathematically specify computational problems. Such specifications should be developed in any revision.

  We reformulated our problems in Section 3 in a mathematical representation by specifying the inputs and outputs.

- Sixth and finally, I never saw a mathematical definition of the neighborhood effect, so it was never clear what it really is; this should be fixed in any revision.

We define the neighborhood effect in lines 10–12. Mathematically, as we describe in Section 3, the neighborhood effect indicates that the POI recommendation function f depends not only on the user's preference for a POI (captured via v_u and v_l), but also on the neighborhood of the POI (which is factored into ω_u and ω_l).
